# Selected Configuration Interaction Using Time-Evolved Population Statistics

**DOI:** 10.1021/acs.jctc.5c01994

**Published:** 2026-04-22

**Authors:** Tim Weaving, Angus Mingare, Alexis Ralli, Peter V. Coveney

**Affiliations:** † 4919 QMatter, Inc., Office 109, 254 Chapman Rd, Suite 101-B, Newark, Delaware 19702, United States; ‡ Centre for Computational Science, Department of Chemistry, University College London, London WC1H 0AJ, United Kingdom; § Advanced Research Computing Centre, University College London, London WC1H 0AJ, United Kingdom; ∥ Informatics Institute, University of Amsterdam, Amsterdam 1098 XH, Netherlands

## Abstract

Selected Configuration Interaction (SCI) is a method in molecular electronic structure theory that allows for the construction of configuration subspaces adapted to the particular system under study. This adaptability is achieved by guiding subspace construction with respect to a selection criterion designed to identify and retain the most important configurations for an accurate description of the system. Quantum-SCI (QSCI) introduces quantum resources to inform the construction of these subspaces, motivated by the classical hardness of state sampling and system dynamics. As with conventional SCI, the CI routine is still performed classically and is therefore not corrupted by hardware noise; only the subspace *quality* is affected by deficiencies in the quantum hardware. Previous QSCI approaches take the measurements produced by a physically motivated circuit construction, often with a recovery step to mitigate against errors, and form a subspace from the resulting configurations. We propose an alternative approach that is more aligned with conventional selection criteria but attempts to inject classically inaccessible information into the selection step with the aim of enabling new subspace expansion pathways. The approach presented in this work uses the population statistics of a time-evolved quantum state to predict likely single and double excitations away from existing configurations to bias the subspace expansion procedure. Importantly, this occupancy-guided expansion complements rather than replaces the direct inclusion of valid configurations sampled from the time-evolved quantum state, so determinants containing higher-order excitations can still enter the variational space in a single iteration. We also include multireference perturbation theory to capture missed correlations outside the configuration subspace. This is demonstrated on hardware by using 42 qubits of an IQM superconducting device to compute the potential energy curve of SiH_4_ in a 6-31G basis set as the Si–H bonds are stretched. We benchmark against the best-in-class Heatbath CI algorithm to assess the compactness of the resulting wave function.

## Graphical abstract

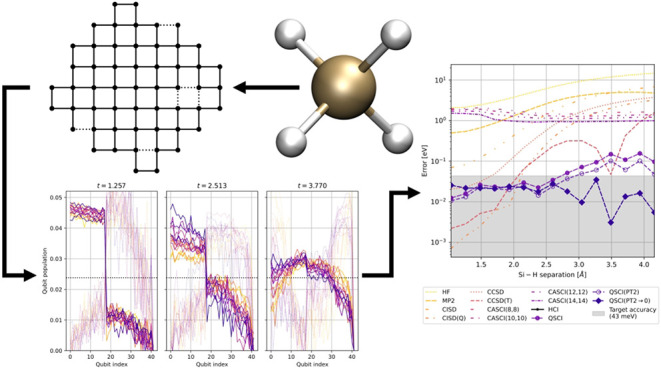

## 1 Introduction

Chemistry has been investigated as an application of quantum computing for over two decades.^1^ The earliest hardware demonstrations appeared in 2010 and studied molecular hydrogen with Quantum Phase Estimation.^2,3^ Since then, we have seen a series of simulations conducted on hardware architectures based on qubit technologies such as photonics, trapped ions, and superconductors.^4–39^ Until the end of 2023, Noisy Intermediate-Scale Quantum (NISQ) simulations of chemistry were dominated by Variational Quantum Algorithms (VQAs), which embed a parameterized quantum circuit within a broader classical optimization scheme.

However, the limited practicality of VQAs meant that NISQ demonstrations had been capped at 12 qubits, as shown in Figure 1. This can be attributed to several real-world limitations, including statistical and hardware noise corrupting the energy estimation procedure, plus the insidious issue of barren plateaus in the optimization landscape.^40–43^ By the end of 2023, it had become apparent that a paradigm shift was needed in our approach to resolving the electronic structure on quantum devices.

**Figure 1:**
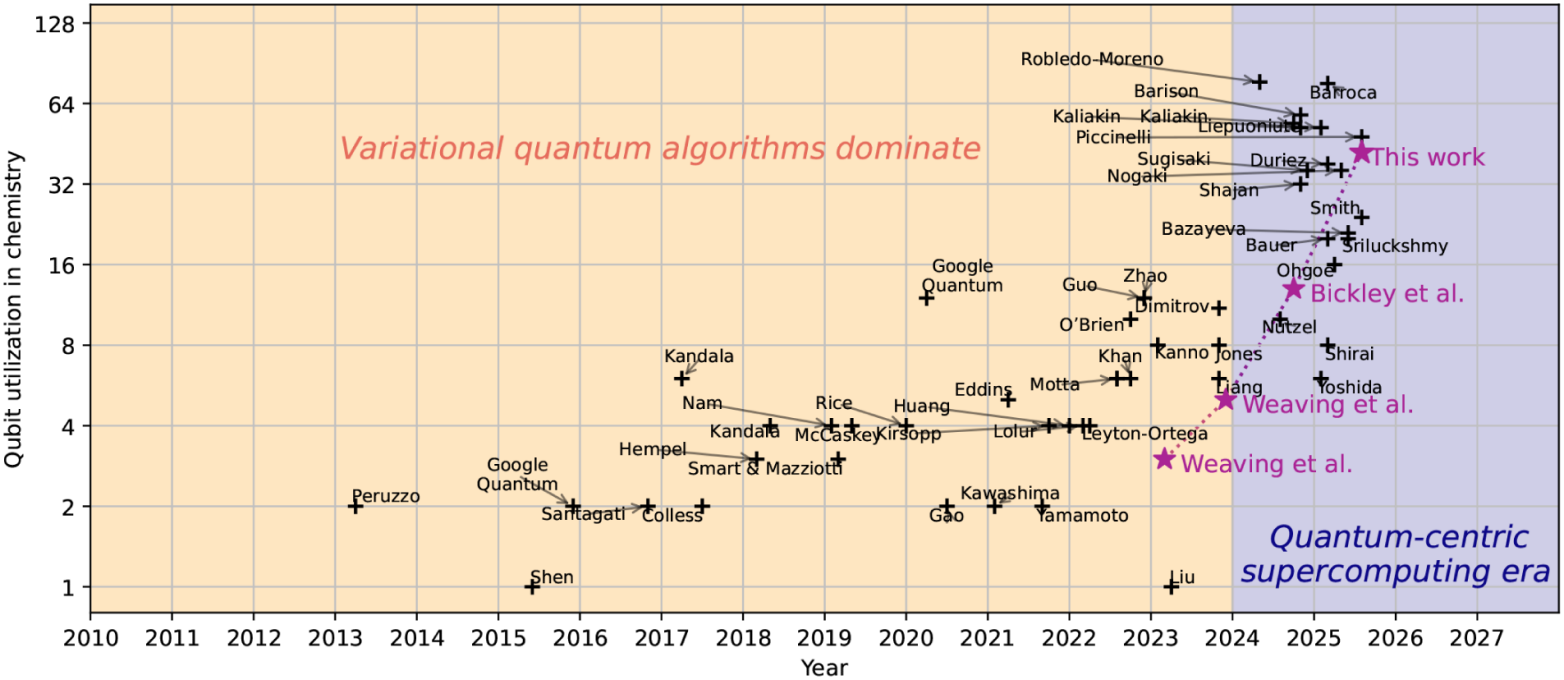
Qubit utilization in chemistry since the beginning of 2013, highlighting the transition from Variational Quantum Algorithms to Quantum-Selected Configuration Interaction techniques after 2024. Dates are taken from the first appearance of the preprint, not the final publication date. Stars indicate work produced by our research group.

A successful framework in conventional electronic structure theory is Selected Configuration Interaction (SCI). Classical SCI algorithms typically expand a configuration subspace by iteratively screening determinants connected to the subspace of the previous step. Determinants that satisfy an importance criterion are appended to the subspace, and the process is repeated, with each step involving the diagonalization of an interaction matrix defined as the projection of the Hamiltonian onto the configuration subspace.

Different SCI methods are distinguished by their importance criteria and are usually designed to minimize energy, which could, for example, weight Hamiltonian matrix elements by wave function coefficients, as with Heatbath CI (HCI),^44–46^ or use more expensive metrics based on perturbation coefficients, such as CI using a Perturbative Selection made Iteratively (CIPSI).^47,48^ The appeal of SCI is in its ability to capture strong correlation effects over a relatively compact wave function representation, while missing dynamical (weak) correlation is optionally treated via multireference perturbation theory.

The question that is currently driving us beyond the era of VQAs in chemistry is whether we can convincingly leverage quantum resources within the SCI framework, an innovation referred to as Quantum-Selected Configuration Interaction (QSCI)^49,50^ or Sample-based Quantum Diagonalization (SQD).^51^ It has been known for some time that quantum computers excel at state sampling, while the same is formally hard for classical counterparts.^52–55^ QSCI aims to exploit this fact in the hope of generating subspaces that would be inaccessible to purely classical approaches. We note that state sampling is not a primitive of conventional SCI, but it is still used to motivate the potential of QSCI to explore regions of Hilbert space that classical approaches might otherwise disregard. Since this hybrid methodology can be demanding on the classical compute, it has inspired the term “quantum-centric supercomputing” ^51,56^ and is positioned to leverage recent trends that see Quantum Processing Units (QPUs) colocated with conventional high-performance computing platforms.^57–59^

Unlike VQAs, inherent hardware noise does not directly enter the energy calculation step of QSCI, since this is handled by a classical CI routine; rather, it is the subspace *quality* that is influenced by device errors. QSCI is therefore more robust to noise compared to quantum algorithms that rely on the estimation of observable expectation values. However, similar to VQAs, we still face the challenge of constructing effective ansatz circuits. In fact, this is more nuanced in the QSCI setting, since the classically tuned wave function need not match that prepared on the quantum device. This observation is central to designing QSCI circuits and should be approached in a different way than VQA ansatz.

The earliest QSCI approaches adopted a circuit construction based on the Unitary Cluster Jastrow (UCJ) ansatz^60^ and localized restrictions to fixed hardware topologies (LUCJ).^61^ The UCJ ansatz approximates the generalized unitary Coupled Cluster wave function using $O\left(M^{2}\right)$ parameters, where *M* is the number of spatial orbitals. While the LUCJ-QSCI approach has been implemented successfully up to 77 qubits,^51,62–74^ its suitability as a candidate algorithm for quantum advantage is under question.

The issue is that LUCJ circuits are parameterized and thus conceal a VQA. These can be initialized from cluster amplitudes, but without subsequent quantum optimization, it seems unlikely to provide any benefit over sampling from a classical approximation to the wave function. While subsequent work aimed to prove that sampling from LUCJ circuits can be classically hard in theory,^75^ it has also been demonstrated that the LUCJ circuits seen in the literature to date are amenable to accurate description by tensor networks.^76^ The difficulty with parameter optimization in this setting is that the QSCI energy is not a smooth function of the circuit parameters. Recent work has attempted to use differential evolution to treat the LUCJ circuit as a black-box importance sampler, directly minimizing the QSCI energy via a gradient-free population search and bypassing the need for ill-defined parameter gradients.^74^ Aside from LUCJ, several other variational circuit constructions have been proposed for QSCI^77–81^ but have the same limitations.

Ideally, our QSCI circuits would be mostly parameter-free to be considered truly “post-VQA”. The natural place to look for this is in Hamiltonian dynamics, a workload that is known to be classically hard; moreover, the only essential parameter is time. This approach has not been studied as extensively as LUCJ constructions but has nonetheless been identified as a promising direction.^82–84^ Moreover, time-evolved variants of QSCI have been formalized in the framework of quantum Krylov subspaces, facilitating the derivation of theoretical performance guarantees.^85,86^

The criticisms of Reinholdt et al. frame the central challenges of QSCI nicely.^87^ The first criticism remarks that, if the trial circuit in QSCI aims to reproduce the molecular wave function faithfully, then the measurement overhead becomes prohibitively large due to overwhelming duplication in the sampled electronic configurations. This is more in keeping with the VQA mindset, wherein the ansatz is intended to approximate the true wave function as closely as possible. We can circumvent this critique by noting that the more desirable feature of a QSCI circuit is that it not only be supported over the dominant configurations in the true wave function but also that it produces those samples close to uniformly. The second criticism asserts that, even in the latter scenario where sampling is evenly distributed over the space of important configurations, QSCI will still produce less compact wave function expansions when compared against competitive classical approaches such as HCI.

To abate the above concerns, one could view QSCI more from the lens of conventional SCI. Instead of directly forming configuration subspaces from quantum measurements, possibly after application of some recovery scheme to sanitize samples that fall outside the desired symmetry sector, we can think about SCI screening metrics that incorporate statistical data from a quantum device. This would allow us to leverage the iterative expansion mechanism of conventional SCI, but with importance criteria that benefit from the injection of classically inaccessible information.

In this work, we explore the idea of using qubit population statistics under Hamiltonian time evolution to bias the subspace expansion step in SCI, or in other words, orbital occupancy dynamics. Our intent is not to discard the determinants observed directly in the time-evolved state but to supplement them with a quantum-informed analog of the classical expansion step for an approach that combines the strengths of both methodologies. We present a practical demonstration on superconducting hardware to simulate the SiH_4_ molecule. We describe this system in the split-valence 6-31G atomic orbital basis set, consisting of 42 qubits and beyond the minimal basis sets often used in quantum computing demonstrations. While it is recommended to use at least 6-31G­(d,p) or cc-pVDZ to incorporate polarization functions for this system, it would require 76 qubits in both cases, which is too large for the available IQM Emerald device, with 54 available qubits. We compare our results against HCI on the basis of wave function compactness; while this presents a high bar to exceed, we make headway toward this goal, where further innovations may continue to close this gap until HCI is surpassed by a QSCI-based approach.

In Section 2.1, we introduce the fundamental concepts of SCI methods, with subsequent multireference perturbation corrections included in Section 2.2. In Section 2.3, we then discuss the motivation behind a Hamiltonian time-evolution approach to QSCI, before describing the stochastic circuit compilation implementation in Section 2.4, which was later used for our practical demonstration. Specifically, we employ the qDRIFT technique of Campbell^88^ to construct shallow time-evolution circuits, an approach to QSCI developed concurrently by Piccinelli et al.^86^ Finally, we introduce our new configuration sampling algorithm in Section 2.5 before outlining the implementation details in Section 3 and presenting the SiH_4_ 6-31G example in Section 4.

## 2 Methods

### 2.1 Selected Configuration Interaction

Configuration Interaction (CI) techniques mix electronic configurations (Slater determinants) to capture correlation effects in molecular systems. Given determinants $D=\{\left|\Phi_{k}\right\rangle {\}}_{k=0}^{K-1}$ , where $\left|\Phi_{k}\right\rangle =\left|b\right\rangle \in {\left(C^{2}\right)}^{⊗N}$ for some binary string $b\in Z_{2}^{N}$ , assumed here to be orthonormal, the configuration subspace projection operator is defined as $P:=\overset{K-1}{\underset{k=0}{\sum }}\left|\Phi_{k}\right\rangle \langle \Phi_{k}|$ . In this subspace, the Hamiltonian *H* takes the projected form

$$PHP=\overset{K-1}{\underset{k,l=0}{\sum }}H_{kl}\left|\Phi_{k}\right\rangle \langle \Phi_{l}|$$ 1

specified by a *K*  ×  *K* interaction matrix ***H*** defined by elements *H*_kl_ = ⟨Φ_*k*_|*H*|Φ_*l*_⟩ which may be evaluated efficiently via the Slater–Condon rules.^89,90^ We obtain the eigenvalue equation ***Hv***_*j*_ = ϵ*_j_ **v***_*j*_ which produces eigenstates restricted to the configuration subspace $\left|\Psi_{j}\right\rangle =\overset{K-1}{\underset{k=0}{\sum }}v_{jk}\left|\Phi_{k}\right\rangle$ with energies $\epsilon_{j}\in R$ , satisfying *PH*|Ψ_*j*_⟩ = ϵ_*j*_|Ψ_*j*_⟩. In the more general case that configurations are nonorthonormal, we replace Slater–Condon with the Löwdin rules^91^ and must also consider the overlap matrix ***S*** with *S*_*kl*_ = ⟨Φ_*k*_|Φ_*l*_⟩ to solve the generalized eigenvalue equation ***Hv***_*j*_ = ϵ_*j*_ ***Sv***_*j*_; since we have assumed orthonormality, we have ***S*** as the identity matrix. In this setting, the development of CI techniques comes down to the way in which we choose D .

If we take D to be complete, meaning it contains every combination of *N*_α_,*N*_β_ spin-up/down electrons in *M* spatial orbitals, we get Full CI (FCI)—the exact solution to the electronic structure problem. The issue is that we have |DFCI|=(MNα)(MNβ) and this scales exponentially in the number of electrons and orbitals.

Alternatively, we may restrict ourselves to an active space (*M*_act_,*N*_act_) denoting *N*_act_ ≤ *N* electrons correlated in *M*_act_ ≤ *M* orbitals, in which $D_{\mathrm{CAS}}$ consists of the configurations that are electronically active within this space. Such techniques are referred to as Complete Active Space (CAS) methods, and while the scaling remains exponential, a sufficiently small active space can be chosen to allow for tractability.

By limiting ourselves to polynomially constrained subsets of configurations, we can simplify the problem to scale more favorably. A common choice is to take the configurations that can be obtained via single or double excitations from the Hartree–Fock reference determinant, producing a subset $D_{\mathrm{SD}}$ of size $\left|D_{\mathrm{SD}}\right|=1+\frac{S\left(S+1\right)}{2}$ , where *S* = *N*_α_(*M* – *N*_α_) + *N*_β_(*M* – *N*_β_) is the number of distinct single excitations, which can be solved in $O\left(M^{6}\right)$ using an iterative diagonalization solver such as Lanczos’^92^ or Davidson’s^93^ algorithm.

Some of the most accurate methods in advanced electronic structure theory aim to choose the configuration space in a better-informed way. For example, one can repeatedly subject the system to perturbations and identify determinants that are “important” to diagonalize the problem in a subspace defined by the dominant contributions in the perturbed wave function expansion. This is the approach of CIPSI^47,48^ and falls under the category of SCI methods, which are differentiated by how importance is assigned to configuration candidates.

An SCI technique that has achieved considerable success is HCI.^44–46^ This works by iteratively expanding the configuration space through solving the Schrödinger equation in the current space $D_{j}$ , yielding eigenstate $\left|\Psi_{j}\right\rangle =\underset{\Phi_{k}\in D_{j}}{\sum }v_{k}\left|\Phi_{k}\right\rangle$ , before screening configurations $\Phi_{l}\notin D_{j}$ and retaining those that satisfy |*H*_*kl*_ *v_k_* | > *δ* for some $\Phi_{k}\in D_{j}$ . Concretely, the expanded subspace is

2 $$D_{j+1}=D_{j}\cup \left\{\Phi_{l}\notin D_{j}|\exists \Phi_{k}\in D_{j}:|H_{kl}v_{k}|>\delta \right\}$$

where δ > 0 is a thresholding parameter that controls the overall accuracy.

While the complement space $\overset{―}{D_{j}}$ is vast, when searching over new configurations $\Phi_{l}\notin D_{j}$ we need to consider only those with *H_kl_≠* 0. This constrains the valid candidates to those related by either single or double excitations, a necessary condition for nonzero *H*_*kl*_ since the molecular Hamiltonian is built from one- and two-body terms. Moreover, since the matrix element magnitude |*H*_*kl*_| depends only on the orbital indices *p*,*q*,*r*,*s* in which the configurations differ (the sign being determined by the remaining orbitals that are common between Φ_*k*_ and Φ_*l*_), we can precompute the doubly excited magnitude data ahead of time.^44^ This process is iterated until convergence, followed by an optional multireference perturbation into the full space from the variationally optimized HCI state. As δ → 0, we have ϵ_HCI_ → ϵ_FCI_ from above, since all configurations will eventually be included.

HCI has been applied to challenging electronic structure problems, such as the chromium dimer Cr_2_ close to the basis set limit,^94^ which was later augmented with DMRG data to produce a composite potential energy curve (PEC) for Cr_2_.^95^ The ability of HCI to capture both strong correlation in the configuration expansion stage and subsequently capture the missed dynamical (weak) correlation through the final perturbation step allows the method to produce highly accurate energies, and the computational cost can be controlled with the δ parameter.

### 2.2 Multireference Perturbation Theory

Following a CI calculation, as described in Section 2.1, we obtain approximate wave function(s) $\left|\Psi_{j}\right\rangle =\underset{\Phi_{k}\in D}{\sum }v_{jk}\left|\Phi_{k}\right\rangle$ with energies $\epsilon_{j}\in R$ satisfying *PH*|Ψ_*j*_⟩ = ϵ_j_|Ψ_*j*_⟩, i.e., eigenstates of the projected Hamiltonian *PHP*. While we can target excited states *j* > 0 through this methodology, in this work we are interested in approximating the ground state |Ψ_0_⟩. Correlation effects in the restricted configuration subspace spanned by D are captured exactly; however, all remaining correlation lying outside D is missed. One may introduce interactions outside the subspace through the application of perturbation theory.

The most convenient formulation for the purposes of SCI is the approach of Epstein-Nesbet,^96^ which takes the model Hamiltonian as

3 $$H_{0}=\underset{\Phi_{k},\Phi_{l}\in D}{\sum }H_{kl}\left|\Phi_{k}\right\rangle \left\langle \Phi_{l}\right|+\underset{\Phi_{k}\notin D}{\sum }H_{kk}\left|\Phi_{k}\right\rangle \left\langle \Phi_{k}\right|$$

and perturbation operator *V* = *H* – *H*_0_. We see that model *H*_0_ consists of the full Hamiltonian block inside the configuration subspace and only diagonal elements outside the subspace. By construction, the SCI wave functions satisfy *H*_0_|Ψ_*j*_⟩ = ϵ_*j*_|Ψ_*j*_⟩, i.e., they are appropriate perturber functions.

Following the standard procedure,^97^ the first-order perturbation correction is ⟨Ψ*j*|*V*|Ψ_*j*_⟩ = 0, and the second-order correction is

4 $$\epsilon_{j}^{\left(\mathrm{PT}2\right)}=-\underset{\Phi_{k}\notin D}{\sum }\frac{\left|\langle \Phi_{k}\right|V{|\Psi_{j}\rangle |}^{2}}{\left\langle \Phi_{k}\left|H\right|\Phi_{k}\right\rangle -\epsilon_{j}}$$

yielding $\epsilon_{j}^{\left(\mathrm{SCIPT}2\right)}=\epsilon_{j}+\epsilon_{j}^{\left(\mathrm{PT}2\right)}$ as the multireference perturbation energy. The memory overhead required in computing $\epsilon_{j}^{\left(\mathrm{PT}2\right)}$ is substantial, so much so that a semistochastic approach was introduced in the context of HCI, whereby an unbiased estimator of the second-order energy correction was derived.^45^

In the completeness limit $D\to D_{\mathrm{FCI}}$ , it is clear the model Hamiltonian *H*_0_ will approach the full-system Hamiltonian *H* (in the desired particle sector), so $\epsilon_{j}\left(D\right)\to \epsilon_{j}^{\left(\mathrm{FCI}\right)}$ and consequently, the correction term must decay to zero, $\epsilon_{j}^{\left(\mathrm{PT}2\right)}\left(D\right)\to 0$ . We can use this fact to motivate an extrapolation scheme by studying the decay of perturbation corrections to estimate the FCI energy. This is investigated in Section 4.

### 2.3 Time-Evolved QSCI

A compelling physical motivation for employing time evolution within the QSCI framework arises from its ability to encode spectral information about the molecular Hamiltonian in a dynamically generated subspace. Starting from a reference state |*ψ*_ref_⟩ such as the Hartree–Fock determinant, real-time evolution produces a coherent superposition of the Hamiltonian eigenstates {|Ψ_*j*_⟩}_*j*_. Decomposing a reference over this eigenbasis yields

5 $$\left|\psi_{\mathrm{ref}}\right\rangle =\underset{j}{\sum }c_{j}\left|\Psi_{j}\right\rangle \to \underset{j}{\sum }c_{j}e^{-i\epsilon_{j}t}\left|\Psi_{j}\right\rangle$$

where the expansion coefficients *c*_*j*_ = ⟨Ψ_*j*_|ψ_ref_⟩ and ϵ_*j*_ is the eigenvalue corresponding to eigenstate |Ψ_*j*_⟩. This operation introduces relative phase shifts between eigenstates, thereby enabling the generation of new configurations that are absent in the initial reference state. We can repeat this process for multiple time steps *t* = *t*_1_,...,*t*_*k*_; for example, with a fixed increment τ∈R , we take *t_k_* = *kτ*. The resulting ensemble of sampled configurations is expected to capture the relevant excitations necessary for an accurate energy estimation.

Although real-time evolution does not inherently amplify low-lying eigenstates in the same way that such contributions dominate long-time dynamics under *imaginary* evolution, it nonetheless possesses notable efficacy when initialized from a physically meaningful reference state. This approach has been investigated by Sugisaki et al.,^82^ Mikkelsen and Nakagawa,^83^ Yu et al.,^85^ and Piccinelli et al.,^86^ the latter two of whom formalize it within the framework of a quantum Krylov subspace method.

Classically, a Krylov subspace is constructed from the successive application of the Hamiltonian on a reference state, i.e., span­({|*ψ*_ref_⟩,*H*|*ψ*_ref_⟩,*H* ^2^|*ψ*_ref_⟩,...}), and forms the basis of iterative diagonalization schemes such as Lanczos’^92^ and Davidson’s^93^ algorithms. In the quantum variant,^98,99^ one instead considers repeated application of the real-time propagation unitary *U* = *e* ^–*i* *H*τ^, yielding a subspace of the form

6 $$\mathrm{span}\left(\left\{\left|\psi_{\mathrm{ref}}\right\rangle ,U\left|\psi_{\mathrm{ref}}\right\rangle ,U^{2}\left|\psi_{\mathrm{ref}}\right\rangle ,...\right\}\right)$$

Higher powers of *U* correspond to longer real-time evolution, *U* ^*k*^ = *e* ^–*i* *Hkτ*^. Choosing a final time *T* and some number of steps *K*, we can prepare time-evolved states |*ψ_k_* ⟩ =*e* ^–*i* *Hkτ*^|*ψ*_ref_⟩ that incrementally propagate by τ = *T*/*K* for *k* steps on a quantum device and subsequently diagonalize the system in the resulting subspace obtained from measurements of the state.

While conventional SCI is effective at iteratively generating and selecting important determinants, the use of time evolution on a quantum device provides an alternative avenue that allows for the implicit identification of significant configurations via Hamiltonian dynamics. The real-time propagator facilitates a physically motivated exploration of Hilbert space, evolving the state along trajectories determined by the underlying interactions, and offers a spectrum-aware strategy for SCI.

However, rather than strictly adopting the quantum Krylov approach described above, our work also aims to align with the strengths of conventional SCI. While we can still choose to retain any valid configurations generated through (eq 6) in our QSCI subspaces, we also wish to devise a configuration sampling routine that incorporates the Hamiltonian dynamics to better guide the expansion step in SCI. To this end, in Section 2.5, we detail an approach that aims to leverage the qubit population statistics of time-evolved quantum states.

### 2.4 Stochastic Circuit Compilation

We now turn to the practical implementation of QSCI on quantum hardware. In this work, we use the Hartree–Fock (HF) determinant^100^ as the initial reference state |Φ_0_⟩, which is trivially prepared with a single layer of Pauli *X* gates. Note that for a more sophisticated approach, the reference state can be updated at each time step using the wave function computed from the previous step, thus further refining the circuit. The challenge then is implementing the real-time evolution operator *U* = *e* ^–*iHt*^. While deterministic product formulas such as first-order Trotter decompositions are simple to implement, the circuit depth grows proportionally with the number of Hamiltonian terms *L*.^101^

For smaller systems, most of the molecular integrals are significant, and therefore the number of two-electron terms increases as $L=O\left(M^{4}\right)$ . However, as the system size increases substantially, the occurrence of non-negligible orbital overlaps is drastically reduced, and consequently, the number of relevant two-electron integrals begins to approach $L=O\left(M^{2}\right)$ ^102,103^ (p.  401). Alternatively, this scaling can be reduced via approximate low-rank decomposition methods, such as double factorization^104,105^ or tensor hypercontraction.^106^

Regardless, for larger systems, this remains prohibitive for NISQ devices. We therefore implement the stochastic compilation technique from Campbell, referred to as qDRIFT.^88^ A similar approach leveraging qDRIFT was developed concurrently by IBM, as detailed in the work by Piccinelli et al., which utilized 48 qubits to simulate an active space of the coronene molecule.^86^

In qDRIFT, rather than deterministically simulating each term in the Hamiltonian, the terms are randomly sampled according to the probability distribution defined by their weights. That is, given a Hamiltonian as a weighted sum of Pauli terms, *H* = ∑*_j_h*_*j*_ **σ**_*j*_, defines probabilities *p*_*j*_ = |*h*_*j*_|/λ, where λ = ∑_*j*_|*h*_*j*_| is the *l*_1_ norm of the Hamiltonian. We then randomly sample *N* terms and append $e^{-i\lambda t\mathrm{sgn}(h_{j})\sigma_{j}/N}$ to the decomposition.

To achieve a given target precision ϵ, the required number of samples is *N* = ⌈2λ^2^ *t* ^2^/ϵ⌉; hence, the circuit depth is effectively decoupled from the number of terms in the Hamiltonian and instead depends on its *l*_1_ norm, λ. With that said, in the worst case, λ can depend linearly on the number of terms in the Hamiltonian, *L*; however, the resulting scaling in *L* is still favorable compared with first-order Trotter decompositions. Furthermore, due to the rapid decay of two-electron integrals with spatial separation, this linear scaling is typically not present in molecular Hamiltonians.

For the purposes of QSCI, at each time step, we generate an ensemble of independent qDRIFT circuit instances, each corresponding to a distinct realization of the stochastic evolution operator. Given multiple quantum devices or a sufficient number of qubits to tile several instances across the same chip, these circuits could be executed in parallel. After collating measurements from these batch runs, the resulting bit-strings are aggregated to define a configuration subspace or otherwise subjected to additional classical postprocessing steps, as detailed in the following section.

### 2.5 Configuration Sampling from Qubit Populations

A considerable challenge of QSCI is handling hardware noise, but it is difficult to design quantum error mitigation strategies since there is a highly nontrivial relationship between the measured bit-strings and the final energy, which involves exact diagonalization over the configuration subspace spanned by the measurements.

By contrast, in algorithms such as VQE, where the energy estimate is obtained via Pauli averaging,^4^ there is a more direct relationship with the measurement distribution. As a result, it is better motivated to employ mitigation techniques such as Zero-Noise Extrapolation^37,107,108^ or Echo Verification^109^ since the influence of noise on the statistical estimator of interest can be more readily studied and rectified.

In QSCI, techniques that directly correct or modify the measured bit-strings are applicable, such as Readout-Error Mitigation^110–112^ or noise-suppression protocols, including Dynamical Decoupling^113–116^ and Pauli Twirling.^117,118^ Strategies targeting the mitigation of estimator bias that act by proxy via statistical averages are not as straightforwardly useful in this context. While the above techniques go some way in improving the results obtained from a quantum computer, it is inevitable that hardware noise will still corrupt the device measurements, particularly as we evolve for longer times in QSCI. The problem here is that, for a given electronic structure problem, it is highly likely that the measurements will fall outside the correct particle/spin sector in most cases, even if the circuit itself respects the Hamiltonian symmetries. It is therefore necessary to devise a method of rectifying noisy bit-strings to produce valid electronic configurations that contribute meaningfully to the wave function.^119^

The *configuration recovery* approach taken by IBM, first presented in the work of Robledo-Moreno et al.,^51^ achieves this by probabilistically flipping measured bits toward the classical spin–orbital occupancy distribution of the recovered wave function |Ψ⟩ obtained from the previous round of subspace expansion. For a bit-string ***b***, the probability of flipping bit *b*_σ,_ *_i_* —corresponding to spatial orbital *i* ∈{0,...,*M* – 1} and spin σ ∈ {α,β}—is proportional to the distance $\left|b_{\sigma ,i}-\left\langle \Psi \right|a_{\sigma ,i}^{†}a_{\sigma ,i}\right|\Psi \rangle |$ between the current bit value and the average occupancy of that orbital with respect to |Ψ⟩. This recovery procedure mitigates noise-induced deviations in particle number and iteratively refines a statistical prior over configurations, guiding the sampling process toward physically relevant subspaces.

The approach developed for this work does not use the orbital occupancy distribution of intermediate QSCI wave functions for configuration recovery but rather the noisy qubit populations measured on the quantum hardware. This distribution is used to predict the most likely single and double excitations above dominant configurations in the QSCI wave function from the previous sampling round, generating a new set of configurations that are subsequently screened for inclusion in the expanded configuration subspace. In parallel, any measured bit-strings that already lie in the correct particle sector are admitted directly into the configuration pool before occupancy-guided expansion. Consequently, the present method is not limited to determinants accessible only by single or double excitations from the current SCI space; higher-order excitations can still be included directly from the time-evolved quantum state.

Specifically, for a measurement set $\{b_{k}{\}}_{k=0}^{N_{\mathrm{shots}}-1}$ , we evaluate the probability distribution $P_{\mathrm{occ}}^{\left(\sigma \right)}$ of an orbital χ_*i*_ being occupied,

7 $$P_{\mathrm{occ}}^{\left(\sigma \right)}\left(\chi_{i}=1\right)\propto \frac{1}{N_{\mathrm{shots}}}\overset{N_{\mathrm{shots}}-1}{\underset{k=0}{\sum }}\left\langle b_{k}\right|a_{\sigma ,i}^{†}a_{\sigma ,i}\left|b_{k}\right\rangle$$

We then iterate over the dominant electronic configurations Φ_*k*_ in the previously diagonalized QSCI wave function |Ψ⟩ = ∑_*k*_ *v*_*k*_|Φ_*k*_⟩, for example, those with a coefficient magnitude exceeding some screening threshold, |*v*_*k*_| > ϵ_screen_. Single and double excitations away from Φ*_k_* are then sampled from a probability distribution conditional on the orbital occupancies of Φ*_k_*. We select a single excitation $a_{p}a_{q}^{†}$ from an occupied index *p* with probability $P_{\mathrm{occ}}^{\left(\sigma \right)}\left(\chi_{p}=1|\Phi_{k,p}=1\right)$ to an unoccupied index *q* with probability $P_{\mathrm{occ}}^{\left(\sigma \right)}\left(\chi_{q}=0|\Phi_{k,q}=0\right)$ , so that the overall probability of drawing the index pair (*p*, *q*) is given by $P_{\mathrm{occ}}^{\left(\sigma \right)}\left(\chi_{p}=1|\Phi_{k,p}=1\right)\cdot P_{\mathrm{occ}}^{\left(\sigma \right)}\left(\chi_{q}=0|\Phi_{k,q}=0\right)$ . The excited configuration $\left|\Phi_{l}\right\rangle =a_{p}a_{q}^{†}\left|\Phi_{k}\right\rangle$ is then added to a screening set, and similarly for double excitations $\left|\Phi_{l}\right\rangle =a_{p}a_{q}a_{r}^{†}a_{s}^{†}\left|\Phi_{k}\right\rangle$ from occupied orbital indices *p*, *q* to unoccupied indices *r*, *s.* The probability *P*(Φ_l_) of sampling the configuration Φ*_l_* is therefore as follows:

- Single excitation *σ → σ*, excitation indices *p*, *q* selected with probability:
$$P_{\mathrm{occ}}^{\left(\sigma \right)}\left(\chi_{p}=1|\Phi_{k,p}=1\right)\cdot P_{\mathrm{occ}}^{\left(\sigma \right)}\left(\chi_{q}=0|\Phi_{k,q}=0\right)$$
- Double excitation *σσ → σσ* (same-spin), excitation indices *p*, *q*, *r*, *s* (*p* ≠ *q*, *r* ≠ *s*) selected with probability:
$$\begin{array}{ll} P_{\mathrm{occ}}^{\left(\sigma \right)}\left(\chi_{p}=1|\Phi_{k,p}=1\right)\cdot & P_{\mathrm{occ}}^{\left(\sigma \right)}\left(\chi_{q}=1|\Phi_{k,q}=1\right)\cdot \\ P_{\mathrm{occ}}^{\left(\sigma \right)}\left(\chi_{r}=0|\Phi_{k,r}=0\right)\cdot & P_{\mathrm{occ}}^{\left(\sigma \right)}\left(\chi_{s}=0|\Phi_{k,s}=0\right) \end{array}$$
- Double excitation *σβ → σβ* (opposite-spin), excitation indices *p*, *q*, *r*, *s* (*p* = *q*, *r* = *s* allowed) selected with probability:
$$\begin{array}{ll} P_{\mathrm{occ}}^{\left(\alpha \right)}\left(\chi_{p}=1|\Phi_{k,p}=1\right)\cdot & P_{\mathrm{occ}}^{\left(\beta \right)}\left(\chi_{q}=1|\Phi_{k,q}=1\right)\cdot \\ P_{\mathrm{occ}}^{\left(\alpha \right)}\left(\chi_{r}=0|\Phi_{k,r}=0\right)\cdot & P_{\mathrm{occ}}^{\left(\beta \right)}\left(\chi_{s}=0|\Phi_{k,s}=0\right) \end{array}$$

Note that only single and double excitations are necessary here, as ⟨Φ_*k*_|*H*|Φ_l_⟩ can be nonzero only when the configurations differ in zero, two, or four spin–orbital positions. Once we have sampled a collection of new configurations Φ_*l*_ connected to Φ_*k*_, they are screened based on the metric

8 $$d\left(\Phi_{l}\right)=\left|H_{kl}\right|\cdot P\left(\Phi_{l}\right)$$

and we append the configurations with the greatest score to the growing configuration subspace. This configuration sampling scheme is guaranteed to remain within the correct particle sector and can be systematically improved, for example, with the screening tolerance, the number of sampling rounds, or the number of configurations appended to the subspace at each iteration.

The ranking metric defined in (eq 8) takes inspiration from HCI, where one screens Φ_*l*_ on the criterion that there exists Φ_*k*_ in the current configuration subspace such that |*H_k_*_*l*_ *v*_*k*_| > δ for some parameter δ > 0.^44–46^ By contrast, in our QSCI approach, we effectively replace the wave function expansion coefficient *v*_*k*_ with the probability *P*(Φ_l_) derived from a quantum experiment; however, given that we only consider excitations away from the configurations Φ_*k*_ such that |*v_k_* | > ϵ_screen_, we do implicitly incorporate the coefficient magnitudes into the selection criteria. Accordingly, our implementation can be viewed as replacing HCI’s exhaustive connected-determinant search with a quantum-informed screening heuristic rather than as claiming a broader classical search space than HCI. The potential advantage instead comes from combining this biased expansion with determinants sampled directly from real-time evolution, which may already contain higher-order excitations that HCI would only discover after many iterations. The full process is detailed in Algorithm 1 of Appendix A.

## 3 Implementation Details

In Section 4, we present a practical demonstration of our stochastic Hamiltonian time-evolution approach to QSCI on superconducting quantum hardware, leveraging the configuration sampling scheme from Section 2.5. The device used is the IQM Emerald QPU, offering 54 qubits arranged in a square lattice, as depicted in Figure 2.

**Figure 2:**
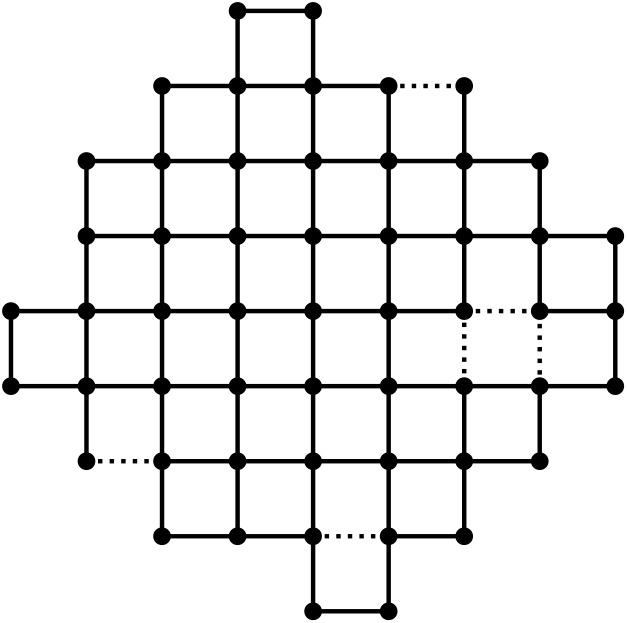
Coupling graph for the IQM Emerald 54-qubit superconducting device. Dotted couplings were not operational at the time of the circuit execution.

We calculate 15 points along the potential energy curve (PEC) produced by varying the Si–H bond length of SiH_4_ 6-31G between 1.040 and 4.158 Å. For each point of the PEC, we perform five time steps in multiples of $\tau =\frac{2\pi }{5}$ and at every step, we generate 50 qDRIFT instances, discussed in Section 2.4, from which 1024 measurement shots are extracted. This amounts to 2.560 × 10^5^ shots per energy estimate and 3.840 × 10^6^ for the whole experiment. In this proof-of-concept study, the five data sets for each time step were collected up-front per bond length, initialized in each instance from the HF reference, and then reused during the subsequent classical sampling and diagonalization procedure. For the SiH_4_ benchmark, this is reasonable as the HF determinant retains sufficient overlap with the ground state across the PEC, but for more strongly correlated systems, an adaptive variant that updates the reference circuit to be subjected to time evolution from the wave function computed in the previous step would be desirable and will be studied in future work.

After collating measurements from the time evolution stage, we enter the configuration sampling phase. Referring to the hyperparameters defined in Algorithm 1 in Appendix A, we set *D*_max_ = 5 × 10^4^ (maximum dimension of the configuration subspace), *N*_rounds_ = 10 (number of sampling rounds per measurement set), *N*_samples_ = 100 (number of samples per screened configuration per round), *ϵ*_screen_ = 10^–2^ (configuration screening parameter), ϵ_WF_ = 10^–5^ (wave function thresholding parameter).

Molecules are built using the PySCF^120^ Python library, and Hamiltonian matrix elements are calculated via the Slater–Condon rules^89,90^ using molecular integrals generated by libcint.^121^ Interaction matrices are solved using the sparse eigensolver in SciPy.^122^ The qDRIFT Hamiltonian sampling functionality is built on symmer^123^ and converted to time-evolution circuits in qiskit.^124^ Quantum jobs are submitted to the IQM Resonance platform for execution on the device.

For the purposes of benchmarking our time-evolved approach to QSCI against a representative suite of classical reference energies, we perform MP2, CISD, CISD­(Q), CCSD, CCSD­(T), and CASCI and HCI calculations to assess the quality of the results. CASCI is evaluated over active spaces (8*o*, 8*e*), (10*o*, 10*e*), (12*o*, 12*e*), (14*o*, 14*e*) and is chosen naïvely around the HOMO–LUMO gap for fair comparison; while these energies can be substantially improved by using natural orbitals, this updates the molecular orbital basis, and thus the Hamiltonian is rotated.

The HCI results are produced by PyCI,^125^ in which the configuration subspace is expanded using a fixed halving procedure applied to the configuration threshold parameter δ introduced in Section 2.1. Initialized with δ = 0.1, the value of δ is repeatedly reduced by a factor of 2 until the subspace reaches the maximum permitted dimension. At each threshold level, the configuration space is enlarged through successive Hamiltonian contractions until no further connected configurations satisfying the threshold criterion are identified.

## 4 Results

In this section, we present a practical demonstration of our QSCI methodology, as detailed throughout Sections 2.1–2.4. The chosen benchmark system is the inorganic silane compound, consisting of four protons surrounding a single silicon atom in a tetrahedral structure, SiH_4_. We represent the problem in the split-valence 6-31G atomic orbital basis set, resulting in a total of 21 spatial orbitals and thus 42 spin–orbitals (qubits) in the molecular Hamiltonian for accommodation on the 54-qubit IQM Emerald superconducting device. The system contains 18 electrons, meaning it is close to the worst-case scenario in terms of the total number of valid electronic configurations for a fixed number of spatial orbitals *M* = 21, specifically (MNα)(MNβ)=8.639×1010 (the binomial is maximized when *N*_α_ = *N*_β_ = *M*/2). While polarized basis sets such as 6-31G­(d,p) or cc-pVDZ would be preferable, they very quickly exceed the size of the chip (76 qubits in that case). The 6-31G­(d) basis set would have required 52 qubits, and although within the 54 qubits available, the offline couplings indicated in Figure 2 would have made circuit construction more challenging.

In the upper row of subplots in Figure 3, we observe how the qubit population, or in other words, the spin–orbital occupancy distribution given in (eq 7), changes as we repeatedly apply the time propagator *e* ^–*iHτ*^ with $\tau =\frac{2\pi }{5}$ to the Hartree–Fock reference for the SiH_4_ 6-31G system on the quantum device. We also plot a transformed version of the distribution *P*_occ_ → *P*_occ_(1 – *P*_occ_), which penalizes orbitals that are either fully occupied or unoccupied, the idea being that this would instead sample preferentially from orbitals of fractional occupancy, where delocalization effects are most prevalent. While we do not use this transformed distribution in the configuration sampling scheme here, it is worth noting that future work could investigate alternative distributions.

**Figure 3:**
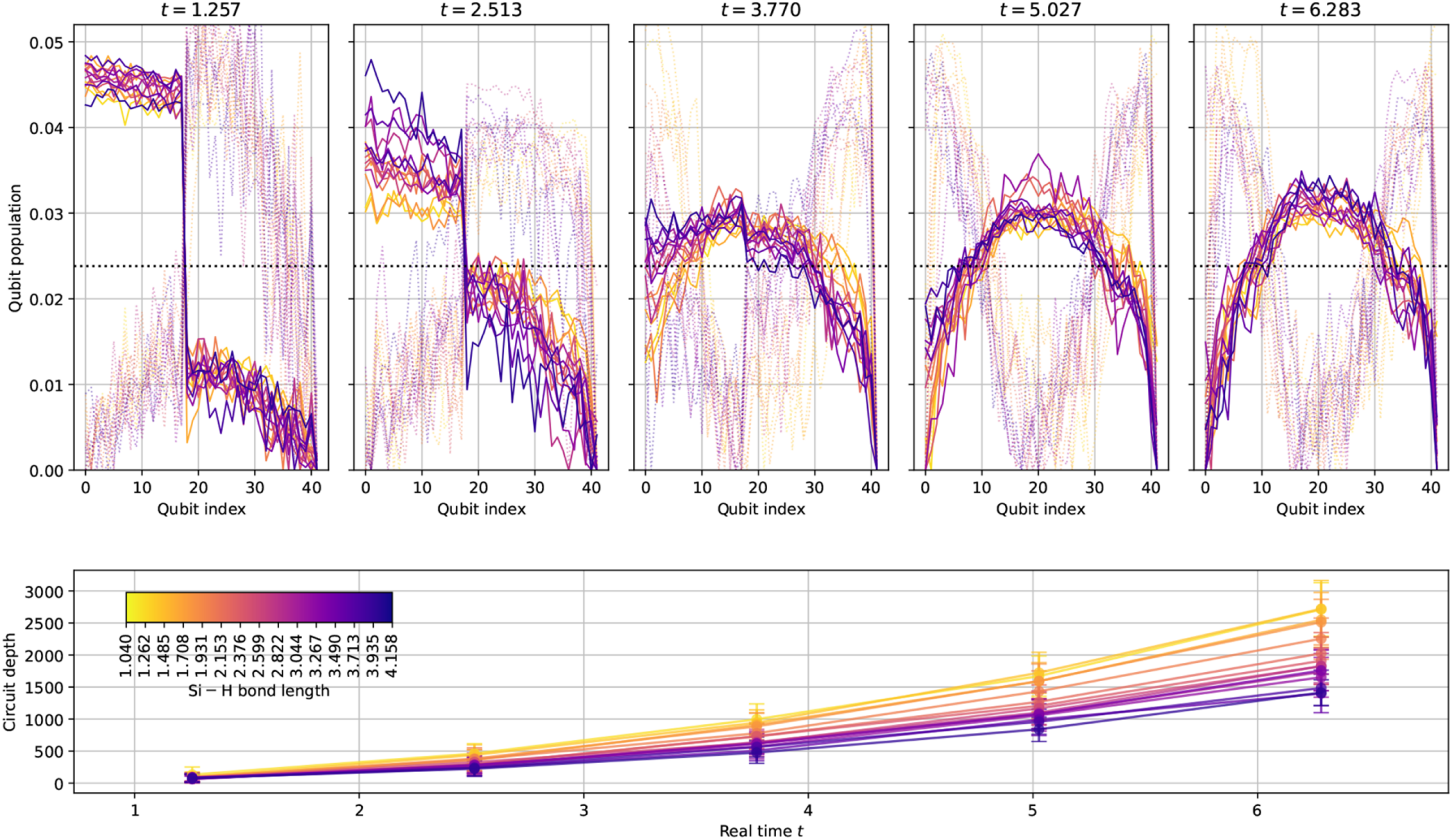
Rectified and normalized spin–orbital occupancy distributions *P*_occ_ for Hamiltonian time evolution applied to the Hartree–Fock reference state on a noisy quantum processor. We also plot *P*_occ_(1 – *P*_occ_) (transparent dotted curves), which penalizes orbitals that are either fully occupied or unoccupied to boost orbitals of fractional occupancy. The time propagation unitary *e* ^–*iHτ*^ was applied up to a maximum of five times for increments of $\tau =\frac{2\pi }{5}$ over different Si–H bond lengths in the SiH_4_ 6-31G system, consisting of 42 qubits. The lower subplot shows the depths of our qDRIFT circuits; note that the circuits for shorter bond lengths have greater depths, which arises due to the corresponding Hamiltonians having larger *l*_1_ norms and thus affects the depth quadratically, as per Section 2.4.

The circuit depths increase according to the qDRIFT algorithm detailed in Section 2.4. Due to its inherent stochasticity, there is substantial variation in the depths across distinct realizations of the time evolution circuit; as seen in the lower subplot of Figure 3, the respective mean 2-qubit gate depths are 43, 161, 361, 640, and 1015 as we step from *t* = 1.257 to *t* = 6.283. For longer evolution times, with consequently deeper circuits, the distribution begins to approach uniform sampling (corresponding with fully depolarizing noise), although it retains a peaked shape. We find the maximum of this peak to align with the Fermi level, between the highest occupied and lowest unoccupied molecular orbitals (the HOMO–LUMO gap). While this could be coincidental and simply an artifact of hardware defects, it is opportune with respect to the configuration sampling scheme detailed in Section 2.5, since this will cause excitations nearer the gap to be selected preferentially over those further away. For shorter time evolutions, in this case up to *t* = 3.770, corresponding with three applications of the time propagator, we are able to discern a jump at the HOMO–LUMO gap rather than the peak observed for later times.

**Figure 4:**
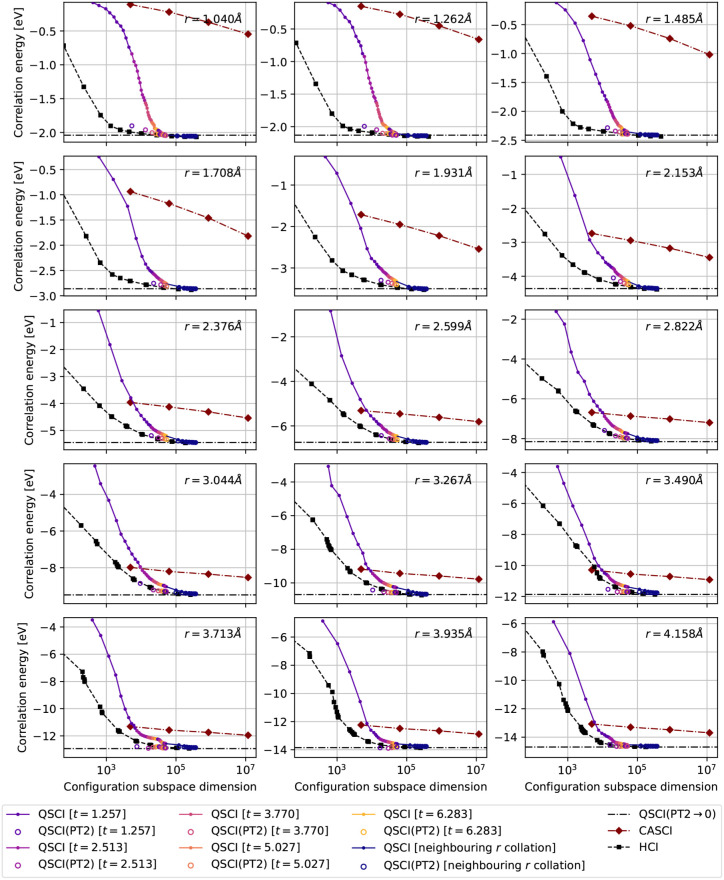
Energy convergence against the size of the configuration subspace for our sampling scheme in time-evolved QSCI, compared with the variational stage of HCI and CASCI calculations over subspaces (8*o*, 8*e*), (10*o*, 10*e*), (12*o*, 12*e*), (14*o*, 14*e*). The first five colors in the QSCI curve relate to different iterations of the sampling scheme and therefore correspond with the five experimental occupancy distributions in Figure 3, excluding the dark blue points which correspond with collating configurations from neighboring points in the PEC to further expand the subspace.

**Figure 5:**
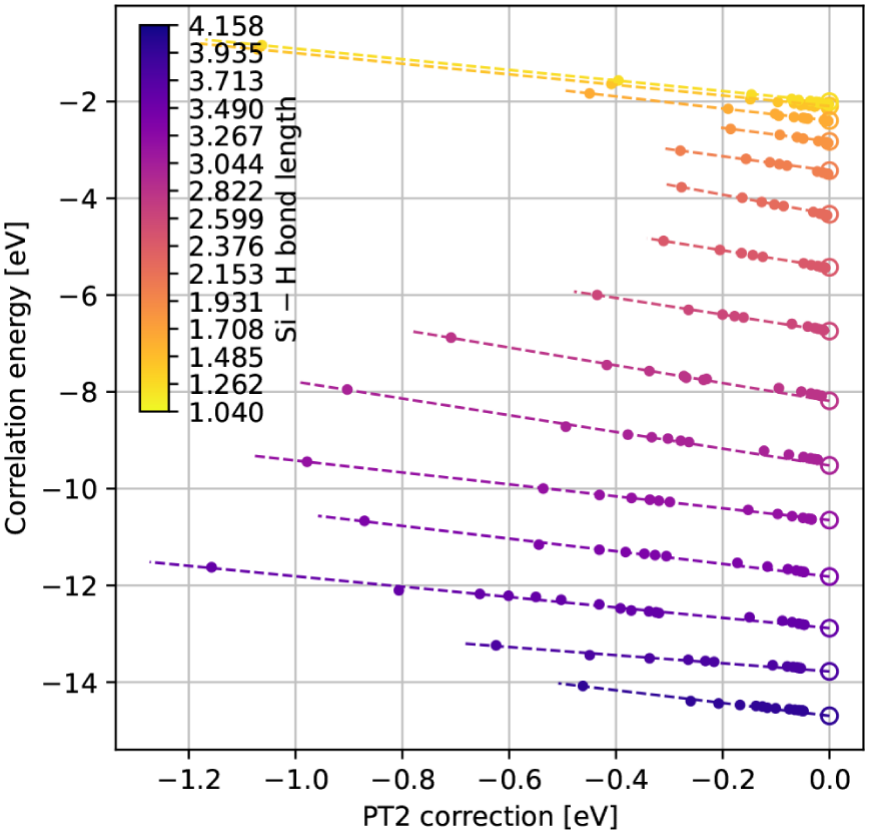
QSCI correlation energy against the second-order perturbation correction. The observed linearity motivates an extrapolation scheme to approximate the FCI energy, where the perturbation correction must decay to zero as the configuration space approaches completeness.

**Figure 6:**
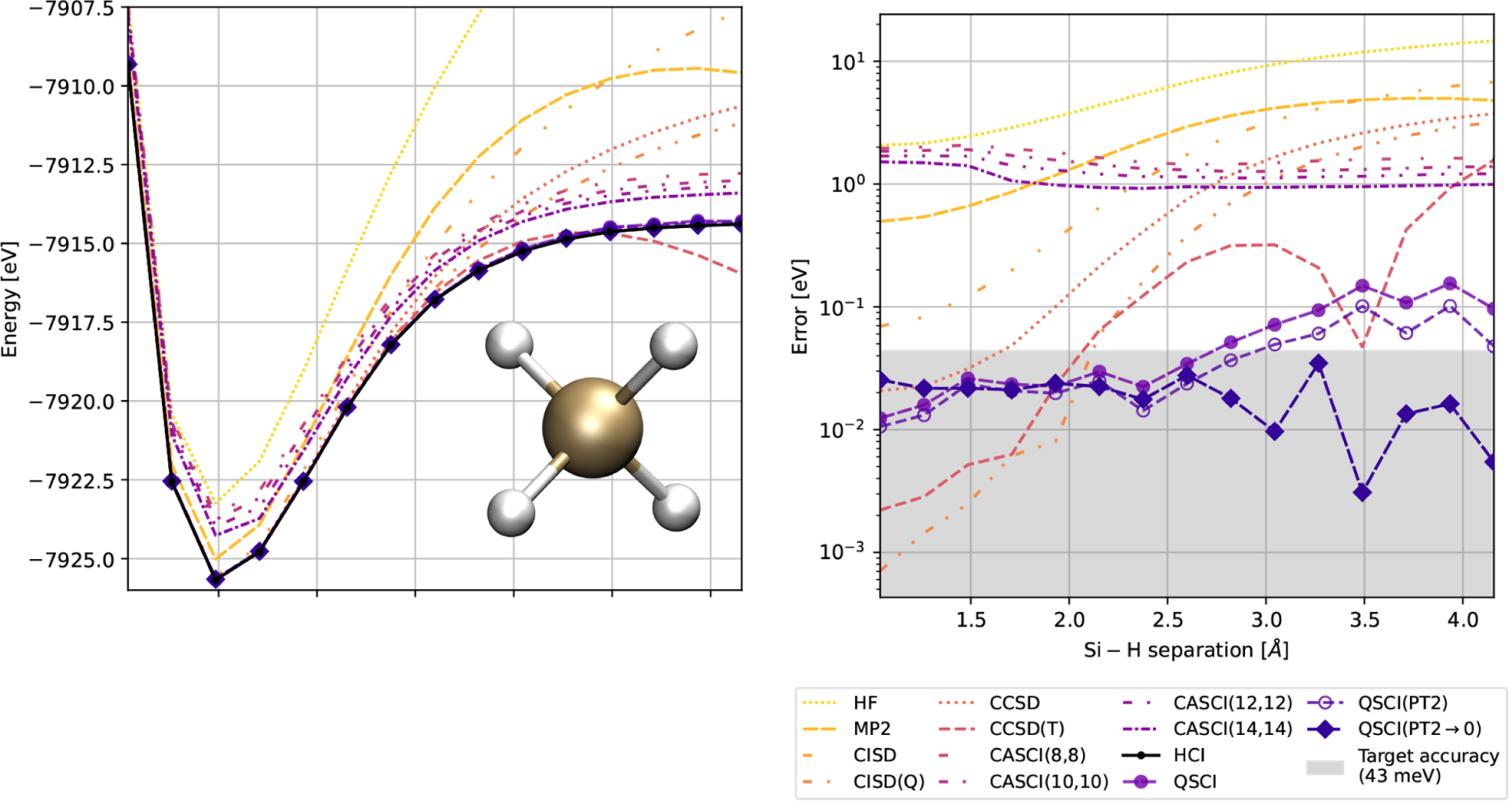
Errors under a stretching of the Si–H bond length between 1.040 and 4.158 Å, comparing various conventional wave function methods against time-evolved QSCI, with and without a multireference perturbation correction, QSCI­(PT2), and an extrapolated zero-perturbation energy, QSCI­(PT2 → 0). Errors are with respect to the HCI energy.

**Table 1:** Summary of Energy Errors with Respect to HCI for Each of the Wave Function Methods in Figure 6 ^a^

Method	Min	Max	Average
RHF	2066.0	14689.7	7423.8 ± 4402.5
MP2	496.6	5000.8	2838.5 ± 1764.0
CISD	69.5	6790.2	2513.2 ± 2368.0
CISD(Q)	0.7	3213.7	972.5 ± 1124.5
CCSD	20.7	3753.2	1295.6 ± 1323.4
CCSD(T)	2.2	1570.9	285.2 ± 417.7
CASCI(8,8)	1450.2	2077.5	1687.8 ± 205.9
CASCI(10,10)	1267.3	1911.8	1484.0 ± 227.7
CASCI(12,12)	1119.8	1701.9	1297.6 ± 212.6
CASCI(14,14)	922.0	1518.6	1067.2 ± 207.2
QSCI	12.4	155.2	60.8 ± 46.9
QSCI(PT2)	10.6	101.5	40.6 ± 28.7
QSCI(PT2 → 0)	3.1	34.8	18.8 ± 8.1

a Values are given in meV, with the target accuracy being 43 meV.

Using the distributions in Figure 3 as the statistical priors that guide the configuration sampling scheme introduced in Section 2.5, we proceed to study the convergence properties of our QSCI algorithm compared against CASCI and HCI. The largest active space (14*o*, 14*e*) of the former consists of 1.178 × 10^7^ electronic configurations. By contrast, the largest subspace sampled in our QSCI routine consists of just 5.625 × 10^4^ configurations. We also continued expanding the QSCI subspaces by collating configurations within an increasing neighborhood around each point of the PEC, up to a total size of 3.656 × 10^5^ and these are denoted by the dark blue points in Figure 4. The central comparison of this work is with HCI, a formidable target for QSCI-based approaches. The converged HCI configuration subspaces have sizes between 1.776 × 10^5^ and 4.767 × 10^5^, similar to our collated QSCI energies.

We see in Figure 4 that HCI more efficiently captures correlation energy early on for smaller configuration subspaces, whereas the converged wave function compactness of QSCI comes very close to HCI. We can look, for example at the subplot for *r* = 3.490 Å where the curves come very close, although HCI manages to retain its edge in this case. Despite this, we are confident that with further enhancements to the QSCI implementation, we will be able to surpass HCI-level wave function compactness.

Also plotted in Figure 4 are the perturbed QSCI­(PT2) energies $\epsilon_{0}^{\left(\mathrm{QSCIPT}2\right)}=\epsilon_{0}^{\left(\mathrm{QSCI}\right)}+\epsilon_{0}^{\left(\mathrm{PT}2\right)}$ , denoted by hollow points. In Figure 5, we plot the QSCI correlation energy, defined as the difference $\epsilon_{0}^{\left(\mathrm{QSCI}\right)}-\epsilon_{\mathrm{HF}}$ with the Hartree–Fock energy, against the second-order correction term $\epsilon_{0}^{\left(\mathrm{PT}2\right)}$ and find a near-linear relationship. As discussed at the end of Section 2.2, as the configuration space expands toward completeness, we must have $\epsilon_{0}^{\left(\mathrm{PT}2\right)}\to 0$ . Therefore, by performing regression, we may extrapolate to the $\epsilon_{j}^{\left(\mathrm{PT}2\right)}=0$ point and take the corresponding value of $\epsilon_{0}^{\left(\mathrm{QSCI}\right)}$ as our approximation to $\epsilon_{0}^{\left(\mathrm{FCI}\right)}$ , denoted QSCI­(PT2 → 0).

Finally, in Figure 6, we compare the PEC obtained from time-evolved QSCI, QSCI­(PT2), and QSCI­(PT2 → 0) against the suite of conventional wave function methods listed in Section 3. Errors in the lower subplot are relative to the converged HCI energy. As expected, the single-reference methods RHF, MP2, CISD, CISD­(Q), CCSD, and CCSD­(T) perform poorly in the dissociation limit. While the CASCI curves exhibit a substantial shift in energy compared with HCI, the errors are predominantly uniform across the PEC, particularly beyond the equilibrium bond length. Excluding the first three points along the PEC, the CASCI error deviates by just 36.0 meV for (14*o*, 14*e*) and 139.8 meV for the smaller (8*o*, 8*e*) active space, thus providing the correct qualitative description of the Si–H bond stretching process. QSCI tracks closely with the HCI curve, having an average error of 60.8 ± 46.9 meV, which improves to 40.6 ± 28.7 meV after the application of the second-order perturbation correction. The most accurate of our QSCI curves is QSCI­(PT2 → 0), which achieves the target accuracy of 43 meV with respect to HCI throughout the full PEC and has a low average error of 18.8 ± 8.1 meV. In Table 1, we tabulate the minimum, maximum, and average errors for each method presented in Figure 6.

## 5 Conclusion

In this work, we presented a new approach to QSCI that uses the qubit populations of stochastically time-evolved quantum states as a statistical prior in the configuration subspace expansion step in SCI. We demonstrated that this approach can achieve wave function compactness comparable to HCI at convergence, and with continued development, we are hopeful that this formidable target can be surpassed by QSCI. While the configuration selection criterion developed in this work is inspired by HCI in the sense that it also includes Hamiltonian matrix element magnitudes in the screening process, it crucially incorporates dynamical information extracted from a quantum computer to bias a sampling process with respect to distributions that are in principle classically inaccessible. Crucially, this occupancy-guided screening operates alongside the direct inclusion of determinants sampled from the time-evolved state, so the method retains access to higher-order excitations beyond the singly and doubly excited configurations obtained from the classical expansion step.

By aligning QSCI with conventional SCI protocols involving iterative subspace expansion guided by a configuration importance metric, we can still benefit from the strengths of the latter while aiming to enhance the screening process with quantum resources. If leveraged properly, this could offer a way of sidestepping the criticisms of Reinholdt et al., who interrogate (a) the measurement overhead when sampling from faithful reproductions of the wave function, and (b) the challenge of improving upon HCI compactness, even when circuits produce uniform distributions over important electronic configurations.^87^ Instead, we propose to use similar expansion mechanisms as conventional SCI, but guided by statistical priors obtained from a quantum device.

Future research will build on the QSCI methodology presented here, with additional benchmarking over a broad selection of systems. We intend to investigate the impact of different sampling distributions on the performance of our methodology and how distributions can be optimized for specific applications. An especially important next step will be to refine the time-evolved reference state adaptively during the QSCI procedure, rather than relying on data collected upfront, so that the quantum dynamics remain aligned with the improving variational wave function. The ultimate goal for this direction in QSCI research is to surpass the incumbent best-in-class by achieving wave function compactness that exceeds the high bar set by HCI.
